# Supplementary material for: λ/30 inorganic features achieved by multi-photon 3D lithography
Source: Nat Commun. 2022 Mar 15;13:1357. doi: 10.1038/s41467-022-29036-7 (PMC8924217; doi:10.1038/s41467-022-29036-7)
Supplement: Supplementary file 1 — Supplementary Information [file 41467_2022_29036_MOESM1_ESM.pdf]

Supplementary Information

$\lambda/30$ Inorganic Features Achieved by Multi-Photon 3D Lithography

Feng Jin,[1] Jie Liu,[1] Yuan-Yuan Zhao,[2] Xian-Zi Dong,[1] Mei-Ling Zheng,*[1,3] Xuan-Ming Duan*[2]*

[1] Laboratory of Organic NanoPhotonics and CAS Key Laboratory of Bio-Inspired Materials and Interfacial Science, Technical Institute of Physics and Chemistry, Chinese Academy of Sciences, No. 29, Zhongguancun East Road, Haidian District, Beijing, 100190, P. R. China

[2]Guangdong Provincial Key Laboratory of Optical Fiber Sensing and Communications, Institute of Photonics Technology, Jinan University, 855 East Xingye Avenue, Panyu District, Guangzhou, 511443, P. R. China

[3]School of Future Technologies, University of Chinese Academy of Sciences, Yanqihu Campus, Huairou District, Beijing, 101407, P. R. China

Corresponding Authors:

*E-mail: jinfeng@mail.ipc.ac.cn (Feng Jin), zhengmeiling@mail.ipc.ac.cn (Mei-Ling Zheng), xmduan@jnu.edu.cn (Xuan-Ming Duan).

Contents

1. Supplementary Methods and Results

1.1 Supplementary Note 1

1.2 Supplementary Note 2

1.3 Supplementary Note 3

1.4 Supplementary Note 4

1.5 Supplementary Note 5

2. Supplementary References

1. Supplementary Methods and Results

1.1 Supplementary Note 1

(1) Optical setups of the femtosecond laser direct writing (FsLDW) of HSQ with transmitted illumination and reflected light illumination

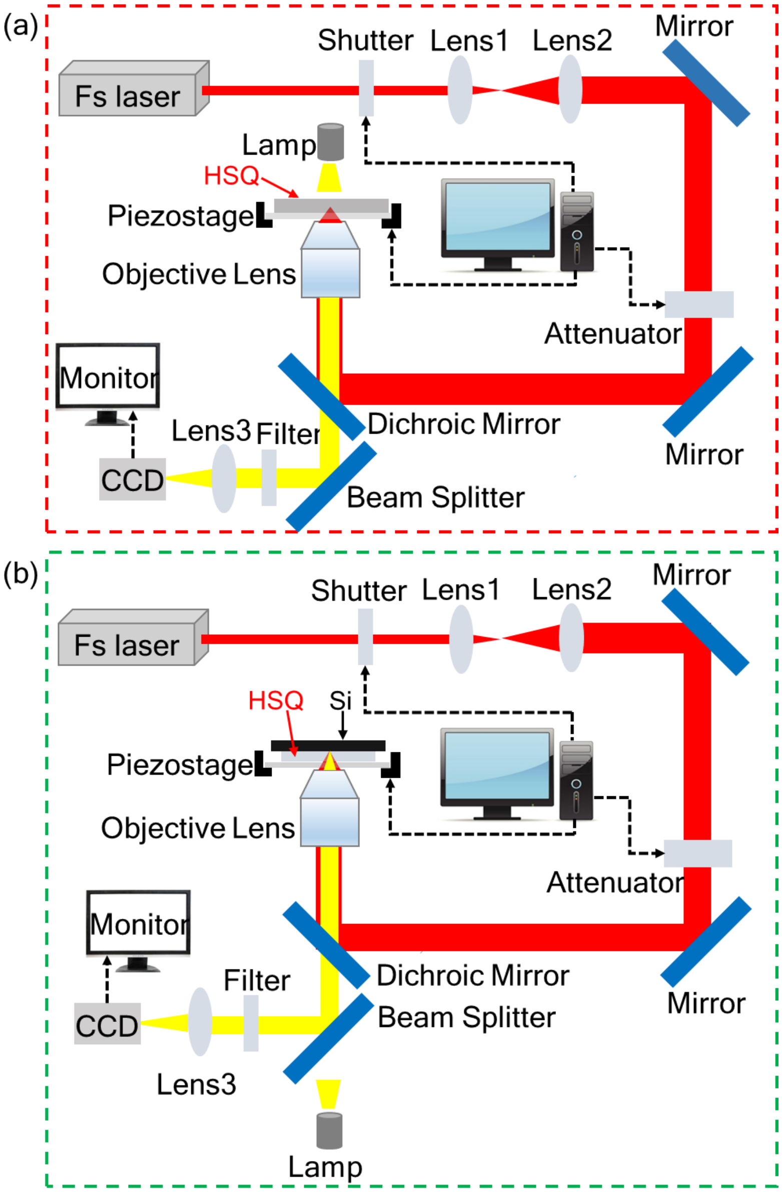

Supplementary Figure 1. Optical setups of femtosecond laser direct writing (FsLDW) of HSQ. **a** Optical setup of FsLDW of HSQ on glass substrate with transmitted illumination system. **b** Optical setup of FsLDW of HSQ on Si wafer with reflected light illumination system.

Supplementary Figure 1 shows the optical setups of the femtosecond laser direct writing (FsLDW) of HSQ with transmitted illumination and reflected light illumination systems, respectively. In brief, FsLDW of HSQ was performed on a fabrication system, which combines an inverted microscope (Olympus, IX 71) and a Ti-sapphire femtosecond laser beam (Tsunami, Spectra Physics) operating at 780 nm, with the pulse width of 120 fs and repetition rate of 80 MHz. The femtosecond laser beam was tightly focused via an oil-immersion objective lens (100 ×, N.A.=1.45, Olympus) into the HSQ film fixed on a 3D piezostage (P-563.3CL, Physik Instrumente), and precisely scanned according to the pre-designed program. A monitor combined with CCD is used to observe real-time fabrication process. As for the fabrication of HSQ on glass substrate, we performed the FsLDW of HSQ on the fabrication system with transmitted illumination, as shown in Supplementary Figure 1a. Nevertheless, when we irradiate the HSQ film on Si wafer with fs laser, due to the blocking of the illumination light by the Si wafer, we conducted the FsLDW of HSQ on the fabrication system with reflected light illumination, as shown in Supplementary Figure 1b.

(2) UV-Vis absorption spectrum of the HSQ film

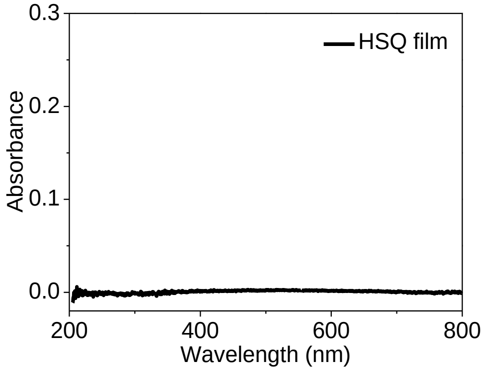

Supplementary Figure 2. UV-Vis absorption spectrum of the HSQ film.

(3) Laser threshold power versus the scanning speed of HSQ by FsLDW

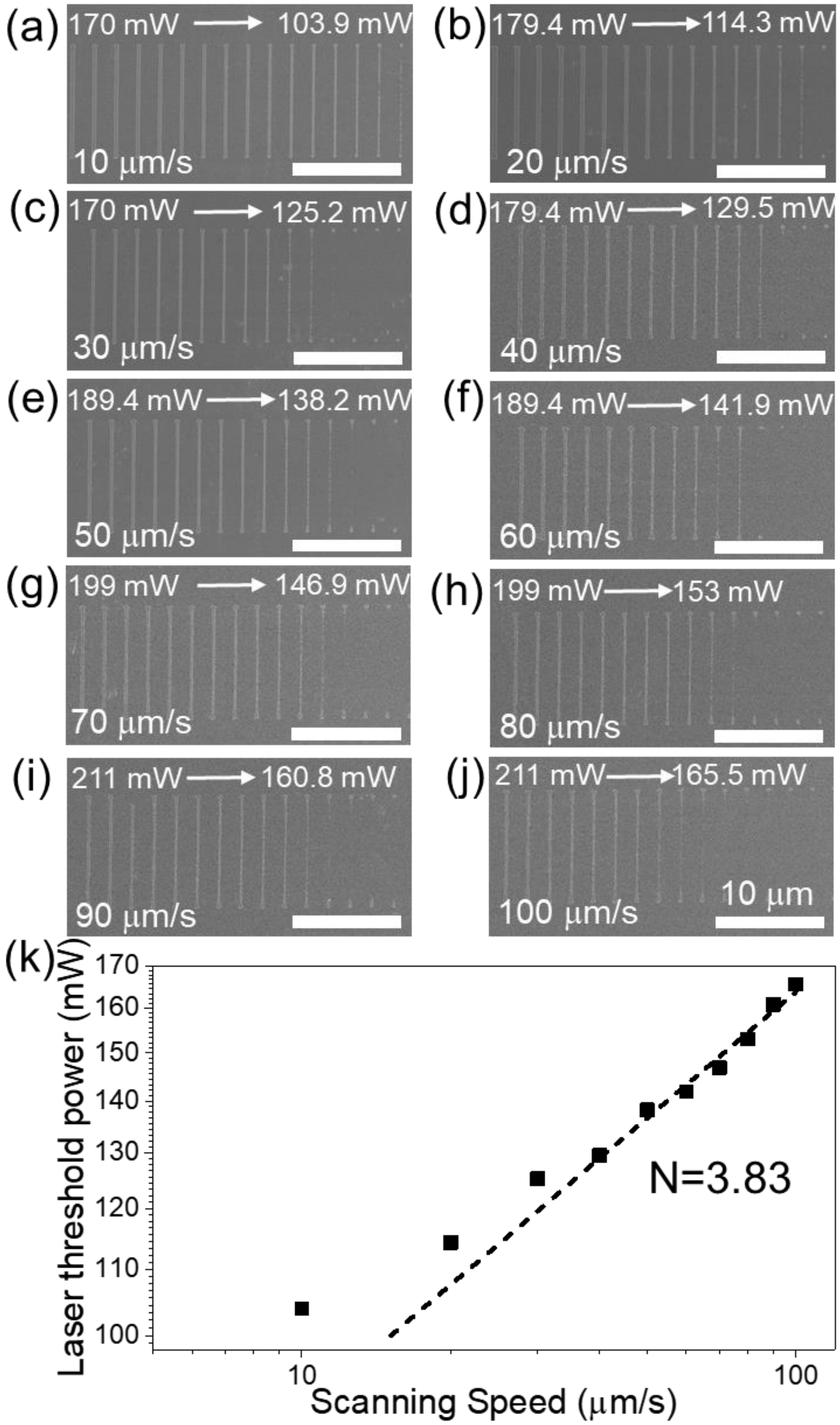

Supplementary Figure 3. Photocuring laser threshold power P_{th} as a function of the scanning speed for HSQ by FsLDW. SEM images of HSQ line array by FsLDW with the scanning speed of **a** 10, **b** 20, **c** 30, **d** 40, **e** 50, **f** 60, **g** 70, **h** 80, **i** 90, and **j** 100 μm/s, respectively. The scale bar is 10 μm. **k** Experimental data and fitting of the laser threshold power versus the scanning speed in HSQ by FsLDW. The square is the experimental data, and the dash line is the fitting result.

We have performed in-site measurement of the nonlinear absorption order in HSQ using the method proposed by Prof. Mueller and Prof. Wegener[1]. The experimental results suggest that multi-photon absorption occurs in HSQ irradiated by a 780 nm femtosecond laser, as shown in Supplementary Figure 3. Supplementary Figure 3a-j present the SEM images of HSQ line array by FsLDW with the scanning speed from 10 to 100 μm/s. For shorter exposure (exposure time smaller than 270 ms, corresponding to a laser scanning speed higher than 50 μm/s), the photocuring laser threshold power versus the scanning speed obey the rule[1-3]:

$$P_{th} \propto C \times v^{1/N} \tag{1}$$

Where P_{th} is the laser threshold power at a given scanning speed, C is a coefficient associated with the characteristic of the photoresist, v is the scanning speed, and N is the nonlinear absorption order in HSQ. For HSQ line array fabricated by a 780 nm femtosecond laser, N is determined to be 3.83 (Supplementary Figure 3k), indicating multi-photon absorption in HSQ by FsLDW. As a result, we depict that FsLDW of HSQ is attributed to multi-photon lithography.

1.2 Supplementary Note 2

(1) AFM images of HSQ nanowires by FsLDW using single-scanning method with different scanning speeds

Morphology of the HSQ nanowires by FsLDW with different scanning speeds are investigated with atomic force microscope (AFM). Supplementary Figure 4 shows the morphology and sectional profile information of the HSQ nanowires by FsLDW with different scanning speeds. According to the AFM results, the width of the HSQ nanowires gradually drops with the increased scanning speed, which agrees well with the SEM measurement (Figure 2c). Nevertheless, the height of the HSQ nanowires keeps almost constant at $\approx$80 nm when the laser scanning speed is ≤ 50 μm/s, then declines to about 30 nm when the scanning speed increases to 100 μm/s. To obtain the detailed morphology information of the HSQ nanowire with the scanning speed of 100 μm/s, zoom-in AFM image of the HSQ nanowire is shown in Supplementary Figure 4c. The Z-profile of the HSQ nanowire along the white line is plotted in Supplementary Figure 4d, and depicts that the HSQ nanowire possesses full width at half maximum (FWHM) of 39 nm. The FWHM of the HSQ nanowire is similar to the linewidth shown in the SEM image (Figure 2c). The construction of HSQ nanowire with width of 33 nm and height of 30 nm further verify the beyond diffraction fabrication of nanoscale HSQ features.

Supplementary Figure 4e-f would be helpful in explaining the dependence of the morphology of HSQ nanowires on laser scanning speed. As shown in Supplementary Figure 4e, isophotes of light with high numerical aperture (N.A.) objective lens is close to an elliptical profile at the focal spot. Photocuring region of the HSQ is determined by the height (h_{focus}) and width (w_{focus}) of the effective focal spot, and the thickness of the HSQ film (t_{hsq}, about 80 nm), as shown in Supplementary Figure 4f. When the scanning speed is low, the height of the effective focal spot is

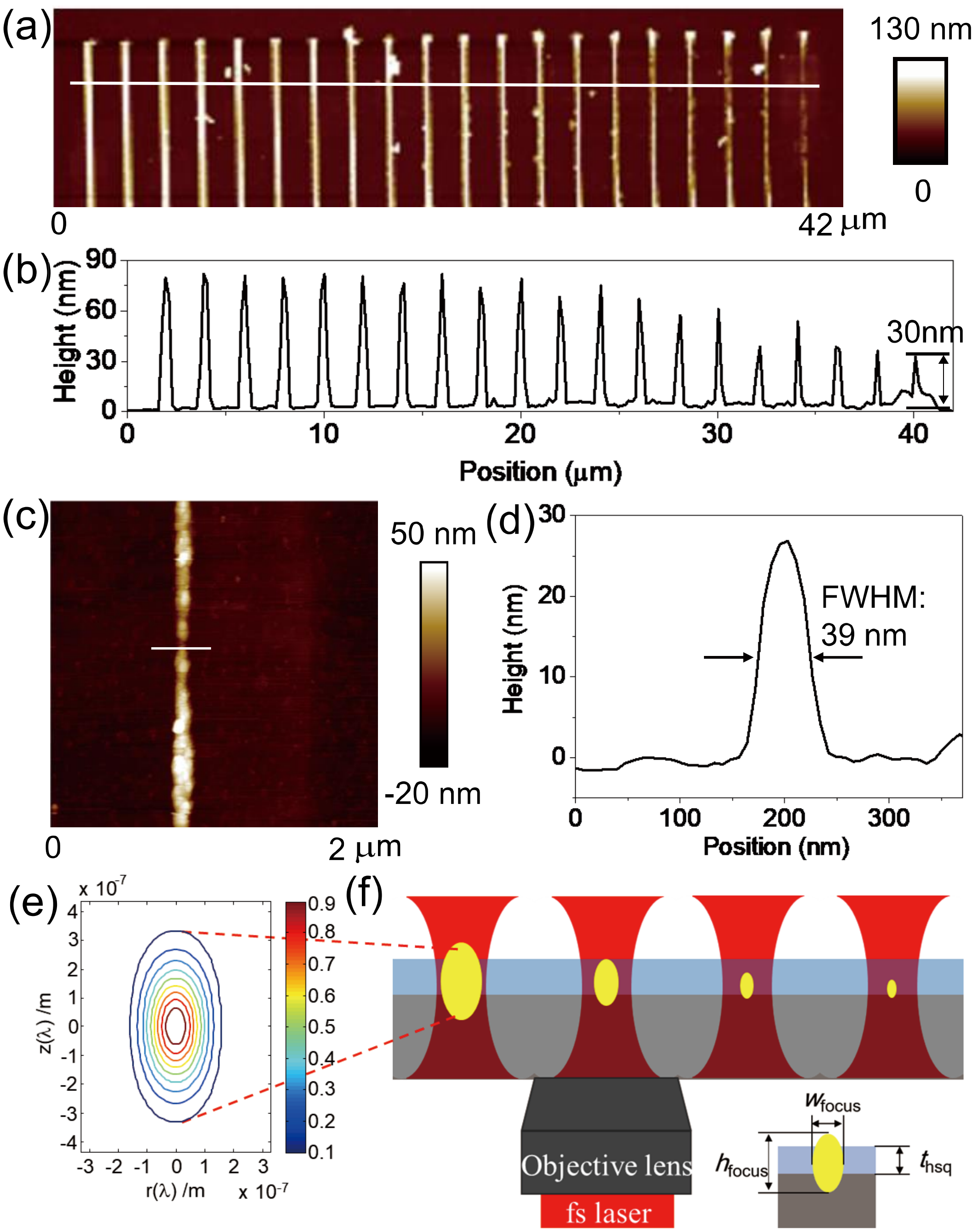

Supplementary Figure 4. a AFM image of the HSQ nanowires fabricated by FsLDW with different scanning speed from 5 to 100 μm/s. **b** Z-profile of the HSQ nanowires shown in **a**. **c** AFM image of the HSQ nanowire fabricated by FsLDW with scanning speed of 100 μm/s. **d** Z-profile of the HSQ nanowires shown in **c**. **e** The isophotes of light intensity are calculated and plotted for the focused 780 nm fs laser pulse. **f** Scheme of the size and position of the fs laser focal spot in the HSQ thin film with increased laser scanning speed.

larger than the thickness of HSQ film ($h_{focus} \geq t_{hsq}$), resulting in the constant height and decreased

width of the HSQ nanowires. When the scanning speed is carefully increased, the height of the effective focal spot is gradually lower than the thickness of HSQ film ($h_{focus} < t_{hsq}$), leading to the formation of HSQ features with nanoscale in both height and width.

(2) Scheme illustration of FsLDW of HSQ features with 33 and 26 nm feature size

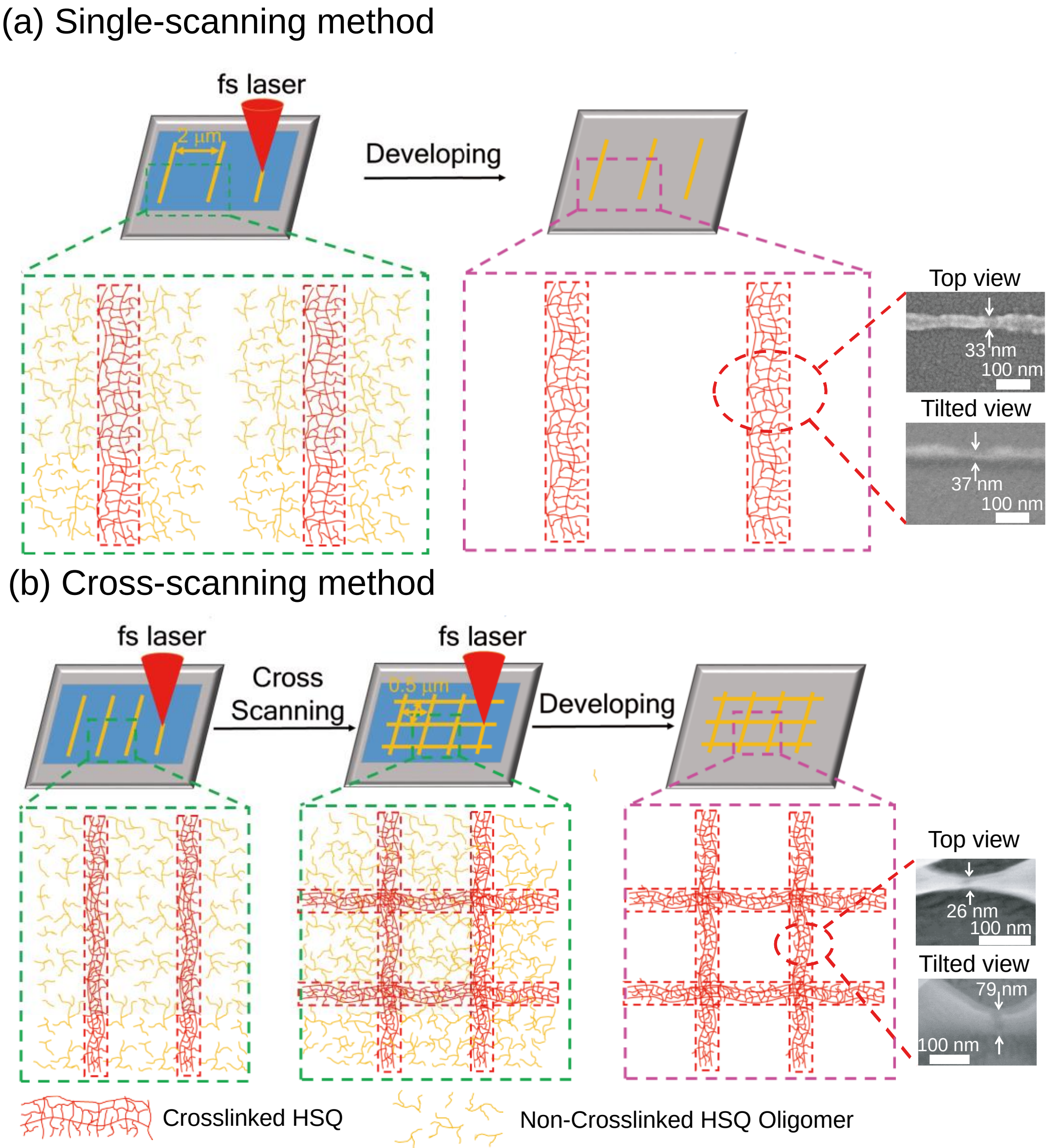

Supplementary Figure 5. Scheme of mechanism of the fabrication of HSQ features by FsLDW with 33 and 26 nm feature size. **a** FsLDW of HSQ feature using single-scanning method with 33 nm feature size. **b** FsLDW of HSQ feature using cross-scanning method with 26 nm feature size.

Supplementary Figure 5 is the scheme to explain the formation of HSQ features by FsLDW with 33 and 26 nm feature size by using two scanning methods. For single-scanning method

(Supplementary Figure 5a), the focused fs laser pulse scans in the HSQ film with 2 μm spacing between adjacent lines. Crosslinking of HSQ occurs at the central region of the focus spot, while the HSQ oligomers appear at the fringe of the focus spot. The former will result in the formation of HSQ features, while the latter will not lead to any feature after development since it's not adequate to support the formation of microstructures. The interference between adjacent lines is neglegible since the spacing of adjacent lines is 2 μm, which is much larger than the laser focus spot. In this case, 33 nm feature size can be achieved due to the threshold effect. Nevertheless, when we perform cross-scanning method with 0.5 μm spacing between adjacent lines (Supplementary Figure 5b), the interference between adjacent lines is no longer negligible. For the laser scanning in the vertical direction, the crosslinking degree of HSQ in the laser exposed region will be enhanced by the existed HSQ oligomers caused by the fringe of the focus spot in the first laser scanning. HSQ features with adequate crosslinking degree can be constructed by reducing the laser intensity closer to the laser intensity threshold. As a result, much smaller region of HSQ can be photocured with laser intensty approaching closer to the threshold, allowing for the formation of HSQ feature with 26 nm feature size.

(3) SEM images of 2D HSQ features by FsLDW employing single-scanning method and cross-scanning method

To collaborate the formation mechanism of the HSQ features by FsLDW through different scanning methods, we studied the relationship between the feature size and the line spacing of 0.5, 1, and 2 μm fabricated by FsLDW with single-scanning method and cross-scanning method, as shown in Supplementary Figure 6. For single-scanning method, the narrowest linewidth of the continuous HSQ nanowires by FsLDW is 53, 58 and 72 nm for the line spacing of 0.5, 1, and 2 μm, respectively (Supplementary Figure 6a, c, and e). The threshold of laser intensity is

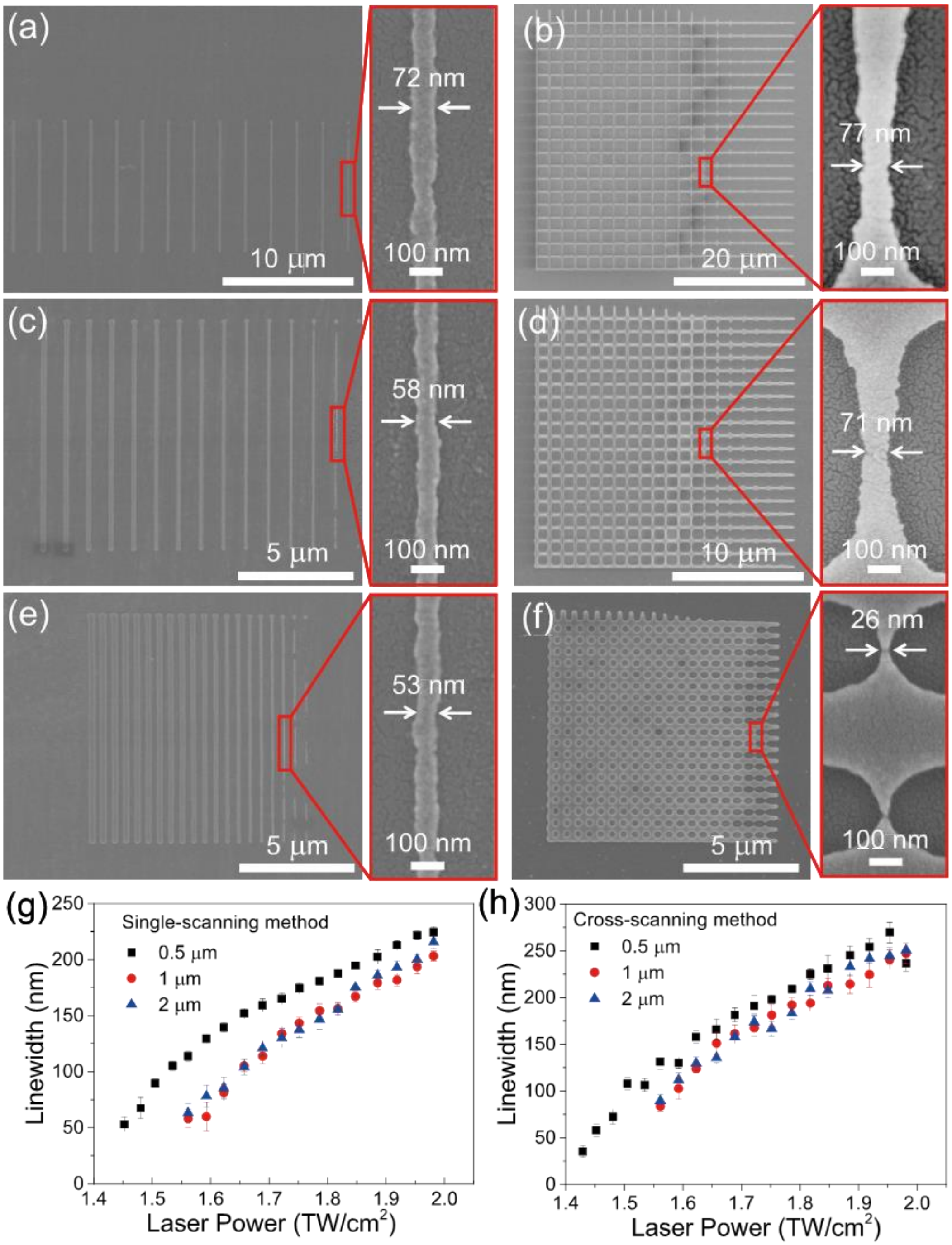

Supplementary Figure 6. Measurement of the feature size of HSQ features fabricated by FsLDW using single- scanning method and cross-scanning method with different spacing. SEM images of HSQ nanowires fabricated by FsLDW using single-scanning method with the spacing of **a** 2, **c** 1, and **e** 0.5 μm, respectively. SEM images of HSQ features fabricated by FsLDW using cross-scanning method with the spacing of **b** 2, **d** 1, and **f** 0.5 μm, respectively. **g** The influence of laser intensity and line spacing on the feature size of HSQ features fabricated by FsLDW using single-scanning method. **h** The influence of laser intensity and line spacing on the feature size of HSQ features fabricated by FsLDW using cross-scanning method.

1.56 TW/cm^2 for the line spacing of 1 and 2 μm, which is larger than that of the line spacing of 0.5 μm, i.e. 1.45 TW/cm^2 (Supplementary Figure 6g). The decrease of the threshold of 0.5 μm spacing is attributed to the interference between adjacent lines. Moreover, the interference between adjacent lines also leads to the bigger linewidth of HSQ for the 0.5 μm line spacing. For cross-scanning method, the feature size of HSQ features is 26, 71 and 77 nm for the line spacing of 0.5, 1, and 2 μm, respectively (Supplementary Figure 6b, d, and f). Compared to the single-scanning method, the feature size of the HSQ feature by cross-scanning method dramatically decreases from 53 to 26 nm for the line spacing of 0.5 μm, and the threshold is decreased to 1.42 TW/cm^2 (Supplementary Figure 6h). The decrease of the feature size is mainly attributed to the interference between cross and adjacent lines.

(4) SEM images of 2D and 3D HSQ features by FsLDW employing cross-scanning method

We performed FsLDW to fabricate 2D and 3D HSQ grids by employing cross-scanning method. Nanoscale features are achieved by precisely tuning the laser intensity with the spacing of 500 nm. During the fabrication of 2D HSQ grids, we fixed the laser intensity in one direction and gradually decreased the laser intensity in the perpendicular direction. As shown in Supplementary Figure 7 a1-a3, 26 nm and 28 nm HSQ features are obtained in the 2D HSQ grid, with the height of 109 and 88 nm, respectively. Furthermore, we depict that nanoscale features can also be constructed in 3D HSQ microstructures, as shown in Supplementary Figure 7b1-b3. Freelying HSQ features with the feature size of 26 and 33 nm are fabricated in the 3D HSQ microstructures by the cross-scanning method. As a result, λ/30 feature size of HSQ by FsLDW is successfully achieved on the substrate, which is significant for the fabrication of nanoscale inorganic features.

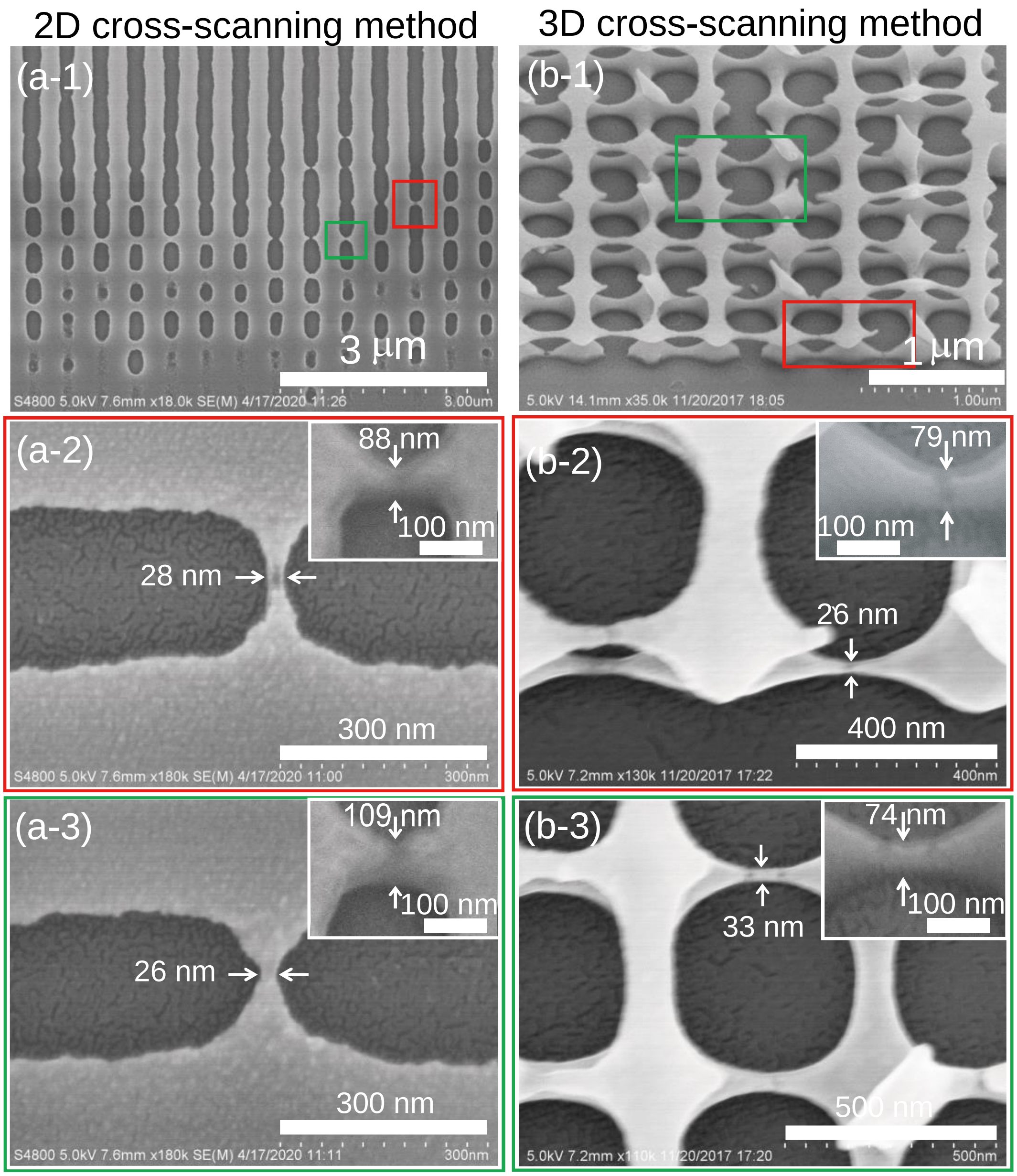

Supplementary Figure 7. **a-1** Tilted SEM image of 2D grid fabricated by FsLDW employing cross-scanning method. **a-2** Magnified SEM image of the region marked by red square. The inset is the tilted SEM image of the HSQ feature. **a-3** Magnified SEM image of the region marked by green square. The inset is the tilted SEM image of the HSQ feature. **b-1** SEM image of 3D HSQ double layered grid fabricated by FsLDW employing cross-scanning method. **b-2** Magnified SEM image of the region marked by red square. The inset is the tilted SEM image of the HSQ feature. **b-3** Magnified SEM image of the region marked by green square. The inset is the tilted SEM image of the HSQ feature.

(5) SEM images of HSQ nanowires by FsLDW employing single-scanning method with different laser intensities

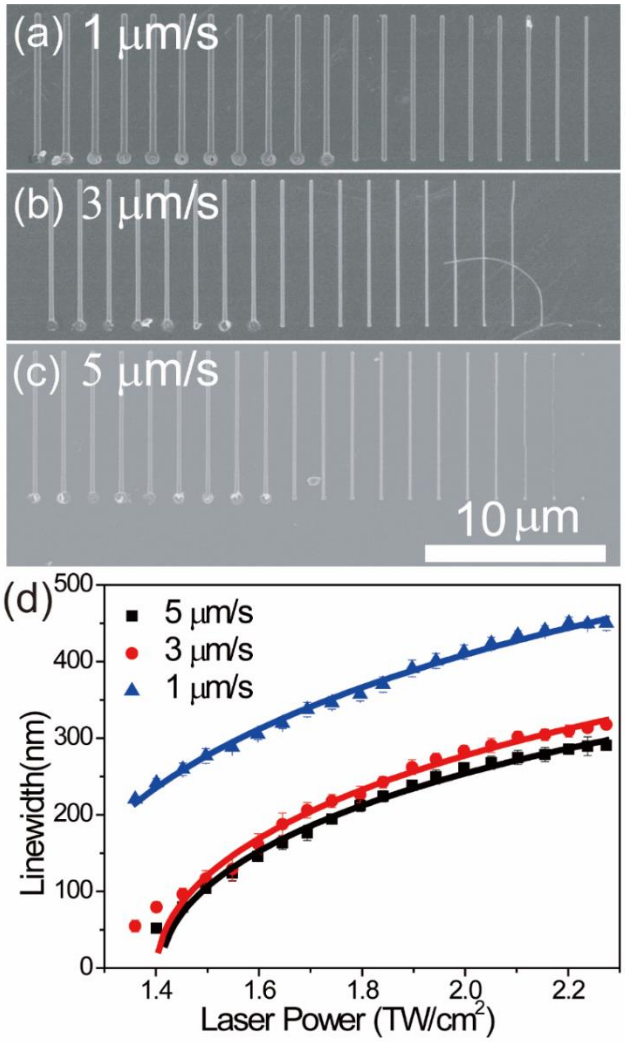

Supplementary Figure 8. Measurement of HSQ nanowires fabricated by FsLDW using single-scanning method with different laser intensities and different scanning speeds. SEM images of HSQ nanowires fabricated by FsLDW using different laser intensities with scanning speed of **a** 1 μm/s, **b** 3 μm/s, and **c** 5 μm/s, respectively. **d** The influence of laser intensity on linewidth of HSQ fabricated by FsLDW using single-scanning method with scanning speed of 1, 3 and 5 μm/s, respectively. The dots are experiment results, and the curves are the fitting results obtained by equation (2).

The fabrication characteristic of HSQ by FsLDW is evaluated via single-scanning method. HSQ nanowires were fabricated by FsLDW using single-scanning method with various laser intensities and different scanning speeds. The influence of the laser intensity on the linewidth is presented in Supplementary Figure 8 for the scanning speed of 1, 3 and 5 μm/s, respectively. With the decrease of laser intensity from 2.27 to 1.4 TW/cm^2, the linewidth decreases monotonically from 450, 318

and 290 nm to 220, 56 and 53 nm for scanning speed of 1, 3, and 5 μm/s, respectively. Further drop of the laser intensity will not allow the formation of HSQ nanowire.

Supplementary Figure 8d illustrates the dependence of the linewidth of the HSQ nanowires on the laser intensity with different scanning speeds. The dependence of the linewidth on laser intensity can be fitted by equation (2), which has been employed to describe the resolution of organic photoresist without photoinitiator through multi-photon ionization (MPI) process[4].

$$D = 2w_0\sqrt{2\ln(\frac{I}{I_{th}})} \qquad (2)$$

Where D is the linewidth of the cured HSQ nanowires, w_0 is the focal radius of the focused fs laser beam, I is the laser intensity of the incident laser, and I_{th} is the threshold laser intensity for photocuring of HSQ. Supplementary Figure 8d shows good agreement of experimental data and fitting curves according to equation (2), indicating a nonlinear multi-photon absorption process of HSQ by FsLDW. As a result, the dependence of linewidth of HSQ on laser intensity can be theoretically predicted, which will facilitate the construction of microstructures with required nanoscale size.

(6) The analysis of the axial size of the 3D double layered HSQ microstructure

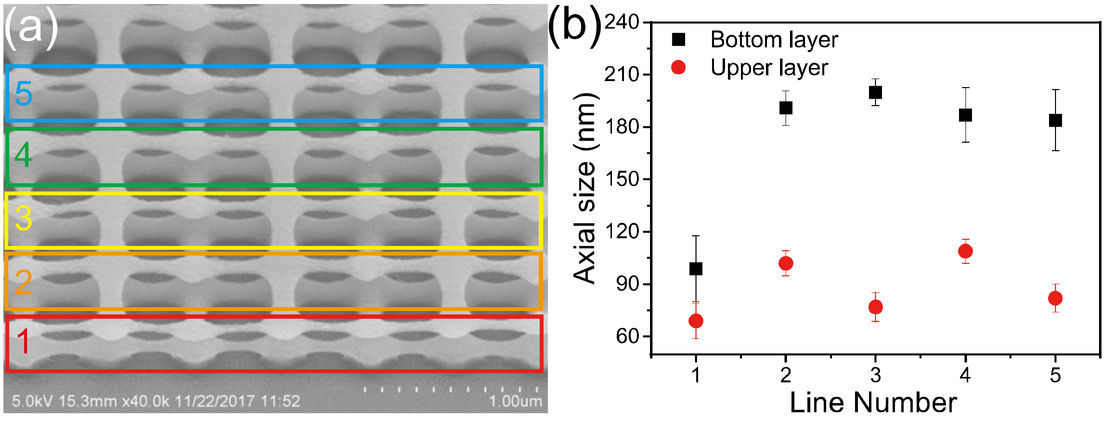

Supplementary Figure 9. a 45° tilted-view SEM image of the 3D double layer HSQ microstructure. **b** The axial size of the upper layer and bottom layer HSQ features.

We analyzed the axial size of the HSQ features for the upper layer and bottom layer in Figure 4g, as shown in Supplementary Figure 9. The upper layer HSQ features are suspended microstrucutres for Line 1-5. The bottom layer HSQ features are freelying on the substrate for Line 2-5, while the bottom layer for Line 1 is suspended microstructures. For Line 2-5, the axial size of the bottom layer HSQ features is around 200 nm, while the axial size of the upper layer HSQ features is around 100 nm. The size of the bottom layer is about two times larger than that of the upper layer, which is mainly attributed to the shrinkage of the features during the development process and surface proximity effects induced by the non-crosslinked HSQ oligomers. The axial size of the HSQ features for Line 1 further verifies the above analysis. For line 1, the axial size of the upper layer HSQ features is around 70 nm, while the axial size of the bottom layer HSQ features is around 100 nm. The axial size of the bottom layer for Line 1 is similar to that of the upper layer for Line 2-5, while much smaller than that of the bottom layer of Line 2-5. The difference is mainly attributed to the fact that the bottom layer of Line 1 is suspended microstructures, instead of freelying microstructures. Compared to the suspended HSQ microstructures, the freelying HSQ microstructures are larger due to the suppressed shrinkage during the development process and the surface proximity effects induced by the noncrosslinked HSQ oligomers.

1.3 Supplementary Note 3

(1) 3D woodpile HSQ microstructure fabricated by FsLDW

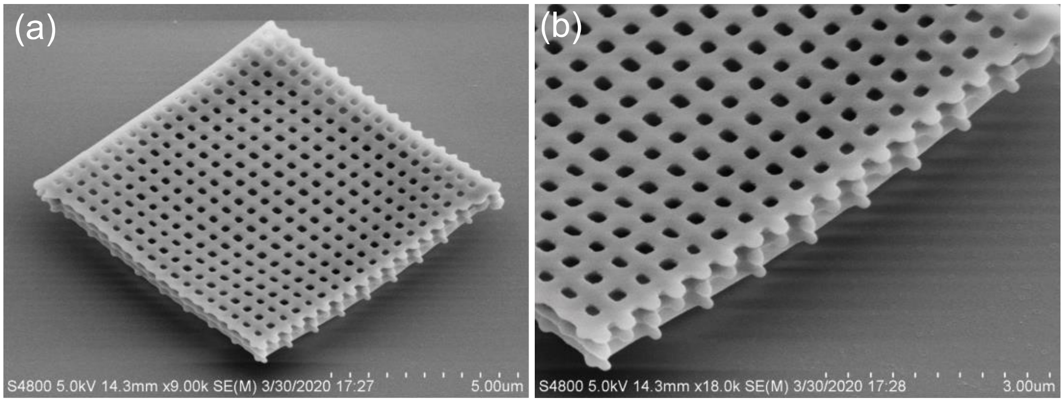

Supplementary Figure 10. **a** Tilted SEM image and **b** Magnified tilted SEM image of 3D HSQ woodpile microstructure fabricated by FsLDW using FOX-16.

3D HSQ feature with woodpile structure is fabricated by FsLDW based on FOX-16. The 3D microstructure is set to have the horizontal periodicity of 1 μm and the vertical periodicity of 0.8 μm, as shown in Supplementary Figure 10.

(2) Deformation of the 3D HSQ microstructure under thermal treatment

Supplementary Table 1. Deformation of the 3D HSQ microstructure under thermal treatment at the temperature of 400, 500, 600, and 700 ℃, respectively.

	nanohole		horizontal		Vertical	
	Size(nm)	Change rate	Size(nm)	Change rate	Size(nm)	Change rate
Pristine (25℃)	149	--	1008	--	439	--
400 ℃	138	-7.4%	1005	-0.3%	386	-12.3%
500 ℃	126	-15.5%	1001	-0.7%	367	-16.5%
600 ℃	119	-20.2%	940	-6.7%	350	-20.3%
700 ℃	destroy	destroy	destroy	destroy	destroy	destroy

(3) SEM images of 3D HSQ microstructures with freelying and suspended features under 400, 500, 600 and 700 ℃, respectively

We investigated the morphology of 3D microstructures after thermal treatment under 400, 500, 600, and 700 ℃, respectively. For the freestanding features, the lateral size increases from 30 to

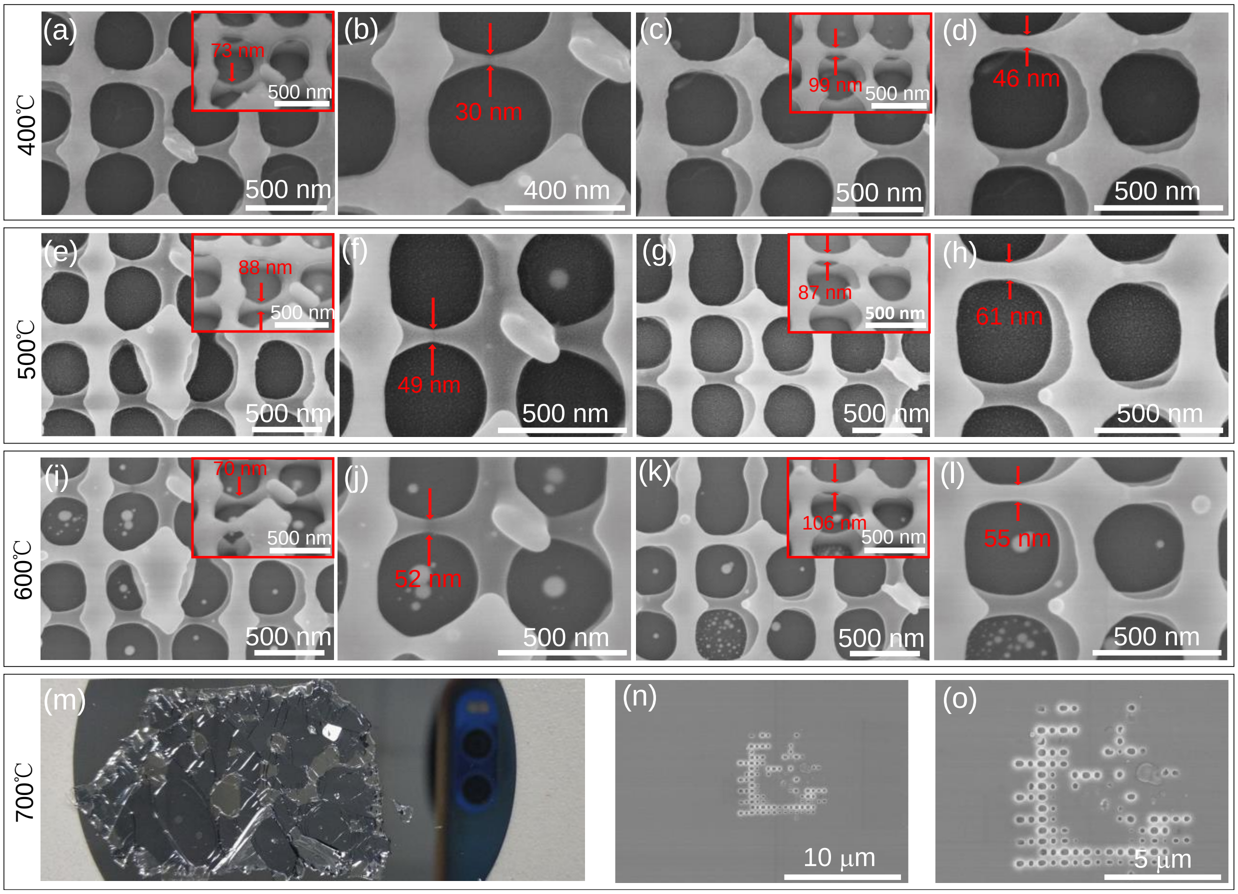

Supplementary Figure 11. SEM images of 3D microstructures after thermal treatment under 400, 500, 600, and 700 ℃, respectively. **a-d**, **e-h**, and **i-l** are SEM images of the microstructure after thermal treatment at 400, 500, and 600 ℃, respectively. The insets in a, c, e, g, i and k are the high-magnification details. **m** Camera photo of the destroyed commercial glass substrate (with 3D HSQ microstructures) fixed on a silicon wafer after thermal treatment at 700 ℃. **n** SEM image and **o** magnified SEM image of the microstructure on the destroyed commercial glass substrate after thermal treatment at 700 ℃.

49, and 52 nm, and the vertical size varies from 73 to 88, and 70 nm after heating at 400, 500, and 600 ℃, respectively, as shown in Supplementary Figure 11a-b, e-f, and i-j. For the suspended features, the lateral size changes from 46 to 61, and 55nm, and the lateral size varies from 99 to 87, and 106 nm when thermal treatment at 400, 500, and 600℃, respectively, as shown in Supplementary Figure 11c-d, g-h, and k-l. Obviously, the 3D nanostructures keep the shape and morphology after heating at 400, 500, and 600 ℃, respectively. Further improving the temperature to 700 ℃, the distortion of the commercial glass substrate occurs, resulting in the destroying of

the HSQ microstructures, as shown in Supplementary Figure 11m-o. It's worth noting that we do not observe the breaking and distortion for the HSQ nanostructures with sub-50 nm feature sizes even up to 600 °C, indicating the potential applications of the HSQ microstructures by FsLDW for harsh environments.

(5) AFM image of HSQ double-layer microstructure with structural colour

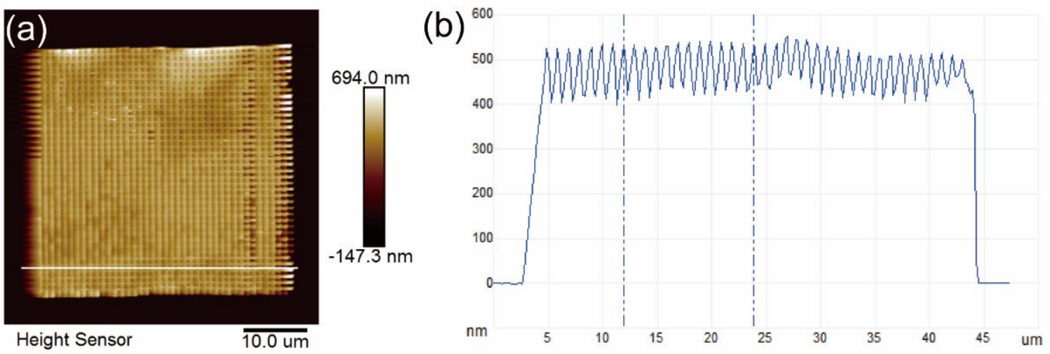

Supplementary Figure 12. a AFM image of the double-layer HSQ microstructure with structural colour. **b** The profile of the HSQ microstructure along the line shown in **a**, exhibiting the height of 500 nm of the HSQ double-layer microstructure.

(4) The refractive index of pristine HSQ, fs laser exposed HSQ, fs laser exposed HSQ after thermal treatment at 400 and 600 °C

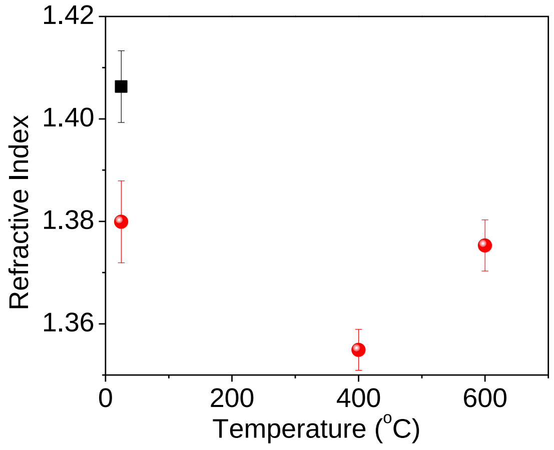

Supplementary Figure 13. The refractive index of the pristine HSQ film (black square), HSQ feature (red ball) structured via fs laser exposure, and structured HSQ features (red ball) under 400 and 600 °C, respectively.

It's important to detect the refractive index of HSQ features by fs laser lithography and further thermal treatment. We have measured the refractive index of the HSQ resin, HSQ features structured via fs laser direct writing, and its deviation after heating (400, and 600 ℃) by using a thin film analyzer (F40-UV, Filmetrics, USA). The measurement procedure is briefly described as follows. *Firstly*, four HSQ films on Si wafer substrates were prepared by spin-coating and evaporation of the solvent. *Secondly*, HSQ micro-squares with the footprint of 50 μm x 50 μm were fabricated on Si wafer via FsLDW. To compare the effect of thermal treatment on the refractive index of the HSQ features, we prepared HSQ micro-squares on three Si substrates by using the same parameters. *Thirdly*, one HSQ micro-square was stored without further treatment after lithography, while another two HSQ micro-squares were treated in a tube oven in Ar atmosphere for 2 hours at the temperature of 400 and 600 ℃, respectively. *Finally*, the refractive index of above samples at 632.8 nm were characterized by using F40-UV. Supplementary Figure 13 plots the refractive index of HSQ resin, structured HSQ via FsLDW, and structured HSQ after thermal treatment at 400 and 600 ℃ for 2 hours, respectively. The refractive index of HSQ film is 1.406, which is similar to the reported value of 1.41[5]. The refractive index of HSQ feature fabricated by FsLDW declines to 1.380. The decrease of refractive index is mainly attributed to the compositional change, i.e. the transfer of cage-network structure[6]. After thermal treatment at 400 ℃, the refractive index of structured HSQ features decreases from 1.380 to 1.355. The drop of the refractive index is ascribed to the film porosity under thermal treatment[6]. Further increase the temperature to 600 ℃, the refractive index of the structured HSQ features increases from 1.355 to 1.375. The increase of the refractive index under higher temperature is mainly due to the disassociation of Si-H bond and film densification[7]. The evolution of the refractive index of HSQ by FsLDW and subsequent thermal treatment is significant for the construction of micro-optical devices.

1.4 Supplementary Note 4

(1) Optical setup for measuring the focusing property of HSQ Fresnel lens

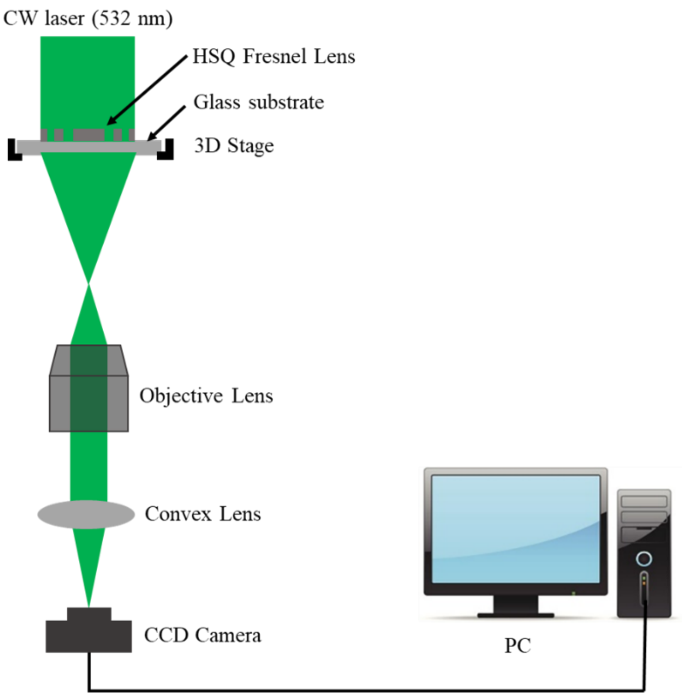

Supplementary Figure 14. Scheme of the measurement setup for characterizing the focusing property of the HSQ Fresnel lens

The optical setup for the measurement of focusing property of the HSQ Fresnel lens is shown in Supplementary Figure 14. In brief, the 532 nm CW laser pulse is attenuated and irradiated on the specimen fixed on the inverted-illumination microscope. The focusing property is characterized by collecting the microscopic images employing a CCD camera connected to a computer. The light distribution of the focus spot is analyzed by using the ImageJ software.

(2) SEM images of HSQ Fresnel lens after thermal treatment

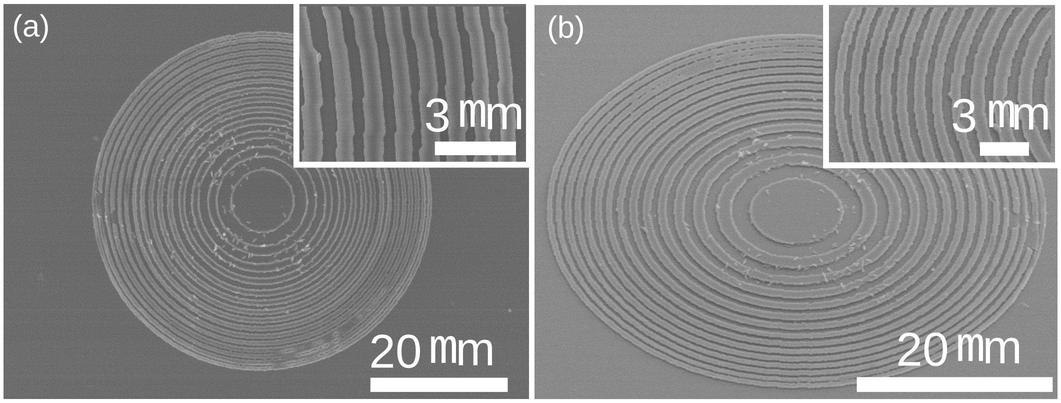

Supplementary Figure 15. SEM images of the HSQ Fresnel lens after thermal treatment. **a** Top view SEM image and **b** titled SEM image of the HSQ Fresnel lens after heating at 400 °C in air for 0.5 hour. The insets are the magnified SEM images in top and tilted view.

(3) Focusing property of the HSQ Fresnel lens after chemical treatment

We investigated the focusing property of the heated HSQ Fresnel lens after being exposed to typical chemical reagents for 1 hour (Supplementary Figure 16). The focus images of the HSQ Fresnel lens exposed to typical chemical reagents are exhibited in Supplementary Figure 16b2-l2, showing focus spots with symmetrically round shape. Supplementary Figure 16b3-l3 plot the full width of half maximum (FWHM) of the optical intensity profile of the Fresnel lens, which varies from 0.8 to 0.83 μm. The change of FWHM after being exposed to the chemicals is less than 3.8 %. It's worth noting that the focusing property of the Fresnel lens did not change much for even being exposed to 98% H_2SO_4, indicating the good chemical resistance of the HSQ microstructure.

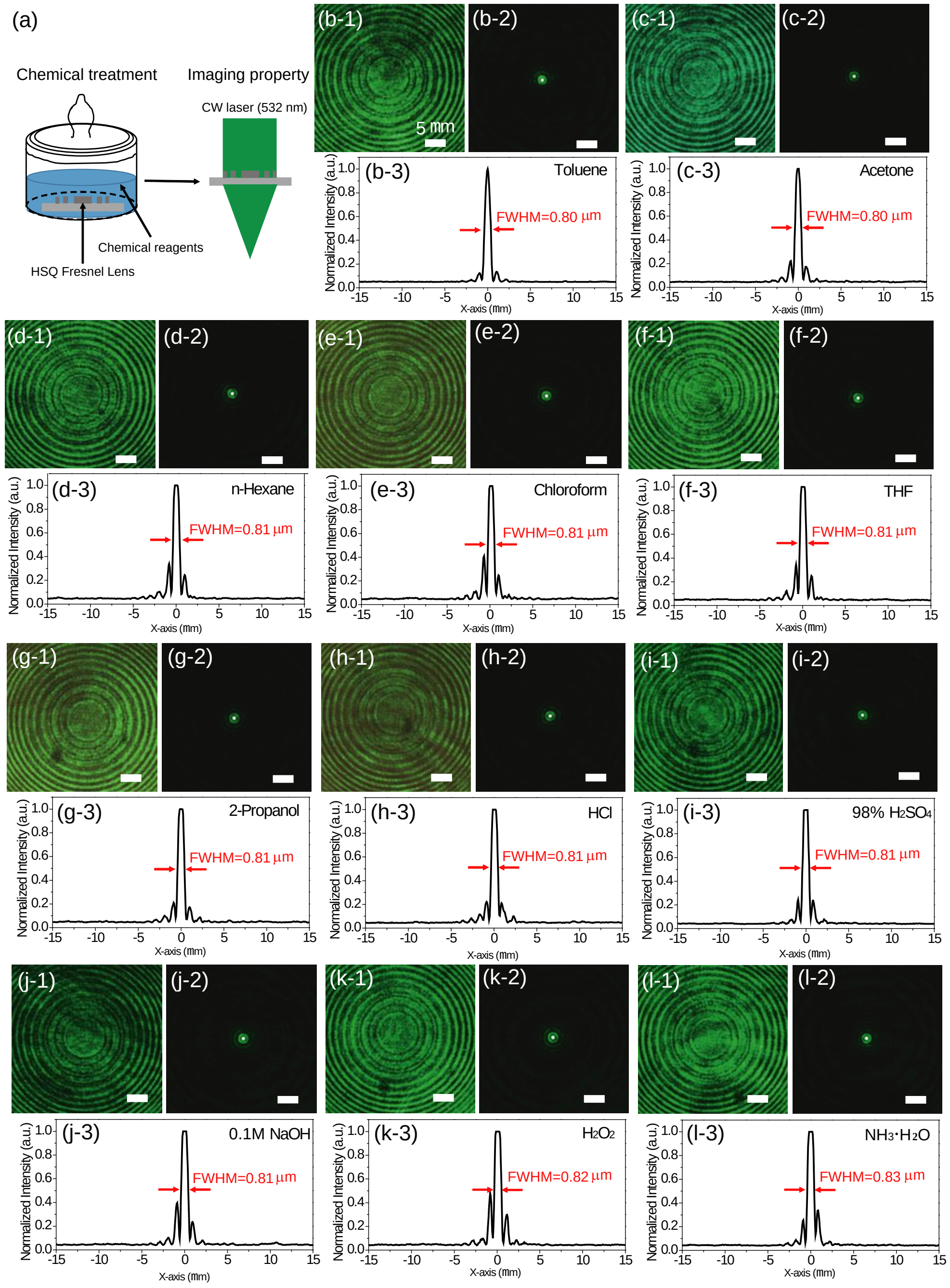

Supplementary Figure 16. Chemical resistance of the HSQ Fresnel lens. **a** Scheme of the focusing property of the HSQ Fresnel lens after chemical treatment. The microscopic focusing images of the HSQ Fresnel lens in the position of **b1-l1** z=0 and **b2-l2** z=44 μm after being exposed to presentative chemical reagents for 1 hour. The scale bar is 5 μm. **b3-l3** The intensity distribution collected along the *x*-axis direction. The chemical reagents include toluene, acetone, n-hexane, chloroform, tetrahydrofuran, 2-propanol, hydrochloric acid (HCl, 37%), sulphuric acid (H_2SO_4, 98%), sodium hydroxide (0.1 M aqueous solution), ammonia solution (25%), hydrogen peroxide (H_2O_2, 30% aqueous solution).

1.5 Supplementary Note 5

(1) Optical property of the HSQ gratings fabricated by FsLDW

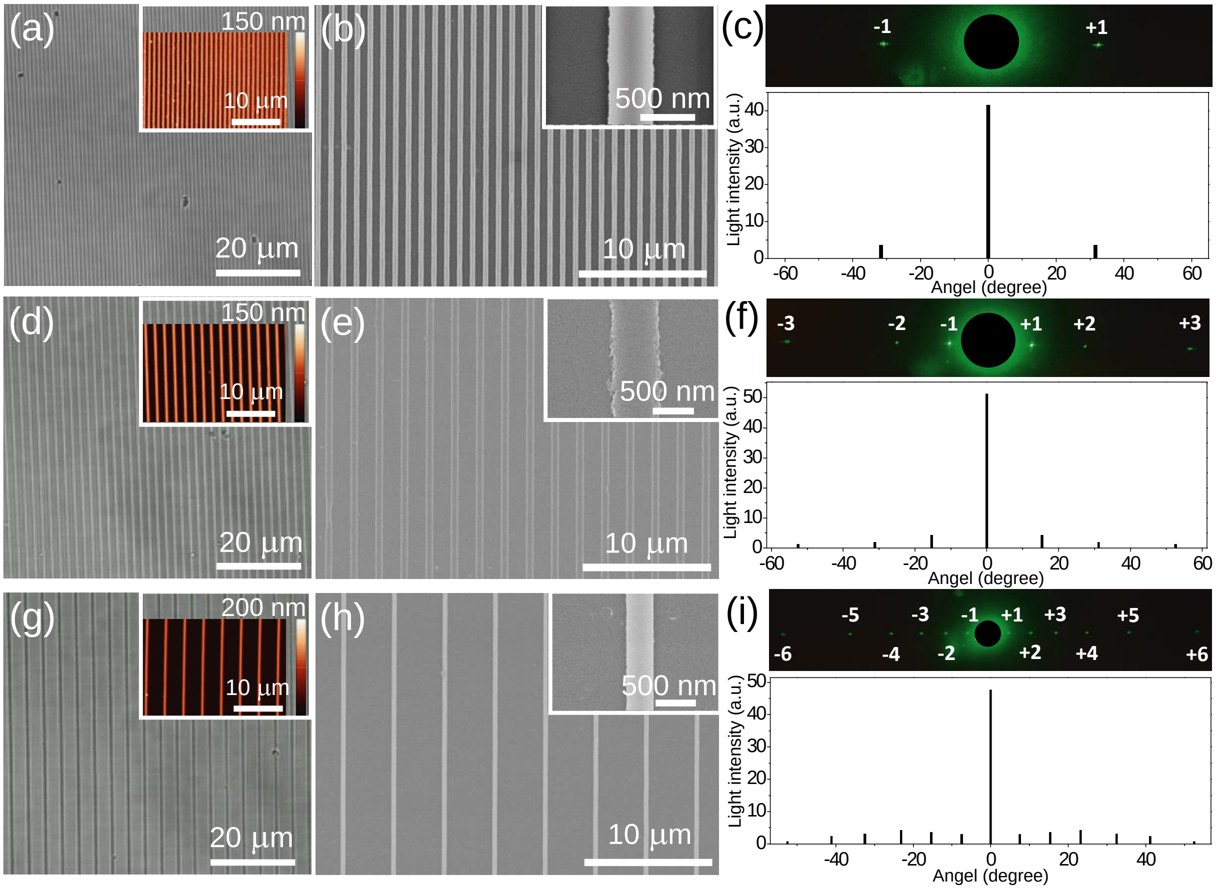

Supplementary Figure 17. The fabrication and diffractive properties of HSQ gratings by FsLDW. Optical microscopy images of the HSQ grating microstructures by FsLDW with the periodicity of **a** 1 µm, **d** 2 µm, and **g** 4 µm, respectively. The insets are the AFM images of the HSQ gratings. SEM images of the HSQ gratings with the periodicity of **b** 1 µm, **e** 2 µm, and **h** 4 µm, respectively. The insets are the magnified SEM images. The diffraction pattern, diffraction intensity and diffraction angel observed in transmission for the HSQ grating microstructures with the periodicity of **c** 1 µm, **f** 2 µm, and **i** 4 µm, respectively. The number refers to the diffraction order of each HSQ grating, and the zero order of the diffraction pattern is covered for clarity.

Moreover, we fabricated diffractive optical device, i.e. gratings with the periodicity of 1, 2, and 4 µm, respectively. The size of each diffractive grating structure was set to 100 µm × 100 µm. Due to the good reproducibility and stability of the photocuring of HSQ by FsLDW, uniform diffractive grating microstructures were fabricated on glass substrates. The uniformity of the HSQ diffractive gratings is verified by the optical transmittance micrograph, AFM and SEM images, as

shown in Supplementary Figure 17a-b, d-e and g-h. The diffraction results of the HSQ gratings with the periodicity of 1, 2, and 4 μm are shown in Supplementary Figure 17c, f and i. In response to the 532 nm laser beam illumination, the grating with the periodicity of 1 μm resulted in first order at 31.6 °, which agrees well with the theoretical value of 32.1° (Supplementary Table 2). As the periodicity of the HSQ grating grew to 2 μm, we observed increased laser diffraction beam spots of the first, second, and third order at 15.3°, 31.1°, and 52.6°, respectively. As for the period width of 4 μm, we recorded the diffracted laser beam spots up to 6th order with the diffraction angles from 7.5° to 52.5°, while the 7th order diffraction spot is too weak to be recognized by the mobile phone camera. The diffraction angles can be theoretically estimated by equation (3):

$$m\lambda = d \sin \alpha \tag{3}$$

In Equation 3, m refers to the diffraction order, λ is the wavelength of incident light, d is the period width of the grating, and α is the diffraction angle for different orders. It is obvious that when the periodicity of the diffractive gratings decreases, the number of supported orders also declines, and each supported order covers a larger diffraction angle. Moreover, the deviation of the diffractive angels between experimental results and theoretical calculation is not more than 1 ° (Supplementary Table 2). The agreement of the diffraction order and the diffractive angle between the experimental results and theoretical calculation verifies high quality of the HSQ gratings fabricated by FsLDW.

(2) Experimental data and theoretical calculation of the diffraction angle of the HSQ gratings

Supplementary Table 2. The experimental data and theoretical calculation of the diffraction angle of the HSQ gratings by FsLDW with periodicity of 1, 2 and 4 μm, respectively.

Grating Periodicity	Diffraction order / Diffraction angel	1st	2nd	3rd	4th	5th	6th	7th
1 μm	Theoretical calculation	32.1°	--	--	--	--	--	--
	Experimental data	31.6°	--	--	--	--	--	--
2 μm	Theoretical calculation	15.4°	32.1°	52.9°	--	--	--	--
	Experimental data	15.4°	31.1°	52.6°	--	--	--	--
4 μm	Theoretical calculation	7.6°	15.4°	23.5°	32.1°	41.6°	52.9°	68.5°
	Experimental data	7.5°	15.3°	23.2°	32.5°	41.1°	52.5°	Too weak

Note: The diffraction angles can be theoretically estimated by equation: $m\lambda = d\sin\alpha$, *m* refers to the diffraction order, λ is the wavelength of incident light (532 nm), *d* is the period width of the grating, and α is the diffraction angle for different orders

(3) Millimeter-sized HSQ grating fabricated by FsLDW

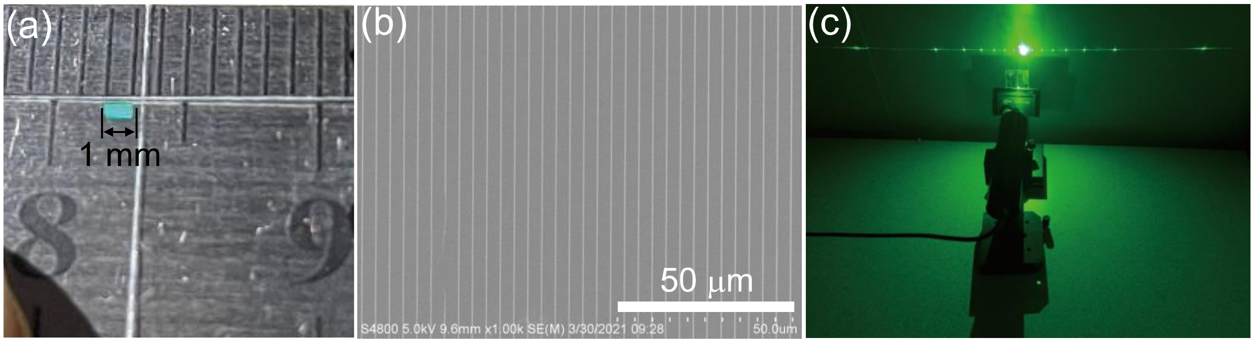

Supplementary Figure 18. a Photo of 1 mm × 0.2 mm HSQ grating structure by FsLDW. **b** SEM image of the HSQ grating structure with the periodicity of 4 μm. **c** The diffraction pattern observed in transmission for the HSQ grating structure.

We further demonstrate the fabrication of HSQ macrostructure by fabricating millimeter-sized HSQ grating structure employing FsLDW. As shown in Supplementary Figure 18a, HSQ grating macrostructure with 1 mm × 0.2 mm has been successfully constructed. SEM image reveals no defect in the uniform HSQ grating structure, in which the periodicity is 4 μm (Supplementary Figure 18b). Supplementary Figure 18c shows the diffraction pattern observed in transmission for

the HSQ grating structures. The diffracted laser beam spots up to 7th order can be well recognized, indicating the high quality of the HSQ grating structure fabricated by FsLDW.

2. Supplementary References

1. Mueller, J. B., Fischer, J., Mayer, F., Kadic, M. & Wegener, M. Polymerization kinetics in three-dimensional direct laser writing. *Adv. Mater.* **26**, 6566-6571 (2014).

2. Yang, L., Münchinger, A., Kadic, M., Hahn, V., Mayer, F., Blasco, E., Barner-Kowollik, C. & Wegener M., On the schwarzschild effect in 3D two-photon laser lithography. *Adv. Optical Mater.* **7**, 1901040 (2019).

3. Yu, H., Ding, H., Zhang, Q., Gu, Z. & Gu, M. Three-dimensional direct laser writing of PEGda hydrogel microstructures with low threshold power using a green laser beam. *Light: Adv. Manufacturing* **2**, 3 (2021).

4. Fischer, J., Mueller, J. B., Kaschke, J., Wolf, T. J. A., Unterreiner, A.-N. & Wegener, M. Three-dimensional multi-photon direct laser writing with variable repetition rate. *Opt. Express* **21**, 26244-26260 (2013).

5. Siampour, H., Kumar, S., Davydov, V. A., Kulikova, L. F., Agafonov, V. N. & Bozhevolnyi, S. I. On-chip excitation of single germanium vacancies in nanodiamonds embedded in plasmonic waveguides. *Light: Sci. Appl.* **7**, 61(2018).

6. Yang, C. C. & Chen, W. C. The structures and properties of hydrogen silsesquioxane (HSQ) films produced by thermal curing. *J. Mater. Chem.* **12**, 1138-1141 (2002).

7. Liou, H. C. & Pretzer J. Effect of curing temperature on the mechanical properties of hydrogen silsesquioxane thin films. *Thin Solid Films* **335**, 186-191(1998).
